# Effect of sodium bicarbonate on the physicochemical properties of fermented rice flour and quality characteristics of fermented semi-dried rice noodles

**DOI:** 10.3389/fnut.2023.1100422

**Published:** 2023-02-16

**Authors:** Wen Xiao, Yuqin Ding, Ying Cheng, Sili Xu, Lizhong Lin

**Affiliations:** ^1^National Engineering Research Center of Rice and Byproduct Deep Processing, Changsha, China; ^2^College of Food Science and Engineering, Central South University of Forestry and Technology, Changsha, China

**Keywords:** sodium bicarbonate, semi-dried rice noodles, pasting properties, rheological properties, cooking properties, textural properties

## Abstract

Considering the effect that fermentation can improve the quality of rice noodles, and given that fermented rice noodles usually have a significantly acidic taste that is not generally acceptable to consumers, this study aimed to neutralize or eliminate the acidic taste of fermented rice noodles by adding sodium bicarbonate, and improve the quality of fermented rice noodles. The physicochemical properties of fermented rice flour and quality characteristics of fermented semi-dried rice noodles were investigated in this study in relation to the addition of sodium bicarbonate (0∼0.5%, w/w). With the increase of sodium bicarbonate addition, the pH value was increased, and lipid and protein content were decreased in rice flour. Meanwhile, thermal properties and farinograph properties showed that the pasting temperature, dough water absorption, dough development time and dough stability time of rice flour increased with the addition of sodium bicarbonate. Pasting properties and rheological properties results showed that a small amount of sodium bicarbonate (0∼0.1%) could increase the pasting viscosity, storage modulus (G’), and loss modulus (G″) of rice flour. Additionally, the hardness and chewiness of semi-dried rice noodles increased with the addition of sodium bicarbonate from 0 to 0.1%. With the addition of a small amount of sodium bicarbonate (0∼0.1%), x-ray diffraction showed that it could increase the crystallinity of semi-dried rice noodles. Low-field nuclear magnetic resonance showed that A_21_ increased, and A_22_ and A_23_ decreased in semi-dried rice noodles. Scanning electron microscope showed that it could enhance the starch-protein interaction and starch-protein formed an ordered and stable network structure. Finally, the principal component analysis showed that the chewiness, texture and eating quality of semi-dried rice noodles were the best with the addition of sodium bicarbonate at 0.1%. This study provides practical value for the application of alkali treatment in rice products and provides a reference for the improvement of related rice noodles products.

## 1. Introduction

Rice noodles are very popular as a traditional staple food in Southeast Asian countries such as China, Vietnam, and Thailand (1). Based on the moisture content, rice noodles can be divided into fresh (> 60%), dry (< 14%), and semi-dried rice noodles (14∼60%) (1). Fresh rice noodles have high moisture content and tend to bind into lumps and break, and have a short shelf life. Dry rice noodles have low moisture content, longer steaming time and cooking loss, and a longer shelf life. Whereas the moisture content of semi-dried rice noodles is in the middle of fresh and dry rice noodles, it has the advantages of a longer shelf life than fresh rice noodles and better taste than dry rice noodles, which is a good choice for consumers’ convenience of consumption (2). Nowadays, the production methods of rice noodles are divided into fermented and unfermented. Studies have shown that the texture and eating quality of fermented rice noodles are better than unfermented rice noodles (3). Through fermentation, the tensile strength (4), elasticity (5), and texture (6) of rice noodles can be improved. However, fermented rice noodles usually have a significantly acidic taste, which is not generally acceptable to consumers.

Numerous starch-based foods, like sticky rice cake (7) and yellow alkali noodles (8), have traditionally undergone an alkali treatment. Alkali treatment has several advantages, such as making it simpler to grind grains and enhancing the appearance, flavor, and texture of starch-foods (8, 9). The most frequent alkalis used in rice goods are sodium hydroxide and calcium hydroxide, which are applied by soaking rice in an alkali solution to change the characteristics of rice flour. According to Lai et al. (10), it was reported that soaking glutinous rice in NaOH alkali solution could increase the peak viscosity and pasting temperature of glutinous rice flour. Pedcharat et al. (11) reported that soaking rice in Ca(OH)_2_ and NaOH alkali solutions, respectively, could be increased the swelling power and solubility of rice flour. Reepholkul et al. (7) reported that soaking glutinous rice in Na_2_CO_3_ alkali solution improved the softness and viscosity of glutinous rice flour. The process of adding the necessary quantity of alkali to change alkali noodle characteristics is known as alkali treatment, and the most popular alkalis used in this process are sodium carbonate, potassium carbonate, and sodium bicarbonate. Kruger et al. (12) and Shiauet et al. (13) reported that the cutting force and tensile strength of extruded noodles increased after adding “Kansui” or a mixture of sodium carbonate and potassium carbonate. Suwannaporn et al. (14) reported that the addition of appropriate amounts of potassium carbonate improved the viscosity, toughness and sensory qualities of noodles. Tao et al. (15) reported that the addition of appropriate amounts of sodium carbonate improved the gluten network and had a significant impact on dough structure, and these changes made the cooking loss and rehydration greater of alkaline noodles. According to Fan et al. (16), the addition of an alkaline solution made from sodium and potassium carbonate increased the cooking time, cooking losses, and hardness and chewiness of noodles. However, most of the studies on alkali treatment in rice products have been done by soaking rice in an alkali solution and then investigating the effect on rice flour. There are few studies on the addition of alkali into rice flour, and there is a need to study the effect of adding sodium bicarbonate on rice flour and rice noodles.

This paper neutralizes or eliminates the acidic taste of fermented rice noodles by adding sodium bicarbonate and also improves the quality of fermented rice noodles. We investigated the effects of different additions of sodium bicarbonate (0∼0.5%, w/w) on the farinograph properties, pasting properties, thermal properties, and rheological properties of fermented rice flour and the cooking properties, textural properties, crystallinity, moisture distribution, and microscopic morphological structure of fermented semi-dried rice noodles. This investigation also provides an opportunity to assess the practical value of rice product applications and to provide a reference for product improvement in related industries.

## 2. Materials and methods

### 2.1. Materials

Indica rice and a strain fermentation solution are both available from Jinjian Rice Industry Co., Ltd., (Hunan, China). The latter is a microbial fermentation system composed of yeast and lactic acid bacteria, with a yeast concentration of 1.0 × 10^4^ to 1.0 × 10^5^ CFU/mL and lactic acid bacteria concentrations of 1.0 × 10^8^ to 1.0 × 10^9^ CFU/mL, respectively. The only food-grade substance employed in the tests was sodium bicarbonate, and all other chemicals, solvents, and reagents were of the analytical grade.

### 2.2. Fermented rice flour

The method of making fermented rice flour as described by Xiao et al. (17). Indica rice was added to the fermenter along with distilled water in the proportion of 1:1.2 (w:v), 4% of the strain fermentation solution, and the fermenter was then placed in a constant temperature incubator (35°C) for 18 h. Following fermentation, indica rice was washed, drained, and crushed. Rice flour was then dried for 12 h at 50°C, after which it was put through 120 mesh sieves and stored at 4°C.

### 2.3. Rice flour sample preparation

Rice flour samples with different additions (0.02, 0.06, 0.1, 0.2, 0.3, 0.4, and 0.5%, w/w) of sodium bicarbonate per 500 g of rice flour were prepared. Rice flour samples were packed in plastic self-sealing bags and stored at 4°C for preparation.

### 2.4. Basic physicochemical composition

According to the AACC methods (18), we calculated lipid(AOAC Method 922.06) and protein content of rice flour samples (AOAC Method 220). From Beijing Solaibao Technology Co., Ltd., the kits for measuring amylose content of rice flour samples were purchased (Beijing, China). We used a pH meter (FE28-Standard, Lichen Instrument Technology Co., Ltd., Shanghai, China) to determine the pH of rice flour samples. We weighed 5.0 g of the sample, added 20 mL of hot distilled water for mixing and shaking, and stood for 5 min to measure the pH value, following the procedure of Yeoh et al. (19).

### 2.5. Farinograph properties

According to AACC method 54-21 (20), the farinograph meter (Micro-dough LAB, Perten Instrument Co., Stockholm, Sweden) was used to ascertain the farinograph properties of rice flour samples. We calculated the dough’s water absorption (WA), dough development time (DDT), and dough stability time were all recorded (DST).

### 2.6. Pasting properties

The pasting properties of rice flour samples were evaluated using a rapid viscosity analyzer (RVA-Super4, Perten Riva Scientific Instruments Ltd., Stockholm, Sweden) by AACC Method 61-02 (20). We created a suspension of 14% rice flour using the sample and distilled water, followed by heating and cooling cycles. The unit of viscosity is CP. RVA characteristic values include peak, trough, final, breakdown, and setback viscosities.

### 2.7. Thermal properties

With a minor modification from the procedures described by Rombouts et al. (21), we used differential scanning calorimetry (DSC 2000, TA Instruments, USA) to determine the thermal properties of rice flour samples. The powder samples were measured into an aluminum crucible, distilled water was added at a ratio of 1:3 (w/w), sealing the container with a tablet press, and the suspension was allowed to equilibrate at 4°C for 12 h. The temperature was raised between 30 and 120 degrees at a pace of 10 degrees per minute. We calculated the onset temperature (T_*o*_), peak temperature (T_*p*_), conclusion temperature (T_*c*_), and gelatinization enthalpy (ΔH).

### 2.8. Rheological properties

Utilizing a rheometer (DHR-2 instrument, TA Instruments, USA), the rheological properties of rice flour samples were evaluated using a modified form of the procedures described by Dorglamud et al. (13). Weighed 5.0 g of rice flour, prepared into a 10% concentration solution by added distilled water, stirred in water bath (95°C) until rice flour paste state, then cooled and measured.

#### 2.8.1. Static rheological

Rice flour paste was laid flat on test bench with the temperature of 25°C and scanning range of 0.1∼200 s^–1^ to determine the trends of shear stress and apparent viscosity of rice flour samples with shear rate.

#### 2.8.2. Dynamic rheological

Rice flour paste was laid flat on test bench with the temperature of 25°C, scanning strain of 2% and frequency range of 0.1∼20 HZ to determine the trends of storage modulus (G′) and loss modulus (G″) of rice flour samples with frequency.

### 2.9. Fermented semi-dried rice noodles

The method of making fermented semi-dried rice noodles as described by Xiao et al. (17). Fermented rice flour (50.0 g), distilled water (62 mL), and different sodium bicarbonate additions were used to make semi-dried rice noodles. Before being extruded into strips by a noodle machine (MS-200, Hualian Shengtong Trading Co., Taiyuan, Shanxi, China), the rice flour was first heated in a water bath (95°C) for 8 min to paste into the dough. This was done to achieve a complete paste. Next, the rice noodles were re-steamed for 3 min and 30 s. Finally, they were aged for 8 h in a room with a constant temperature (90% RH, 25°C). After aging, semi-dried rice noodles were packaged, sterilized for 30 min in hot water (95°C), and then promptly chilled in cold water to stop the growth of bacteria.

### 2.10. Cooking properties

#### 2.10.1. Cooked broken rate

The cooked broken rate of semi-dried rice noodles was calculated using a modified form of the Xu et al. (22) methods. We selected 20 semi-dried rice noodles more than 15 cm in length into a beaker, added boiling water, heated them on an electric stove, and placed them in a test sieve for 5 min after the optimal cooking time (2 min and 30 s, completely softened and no white core of cooked rice noodles). Following the Eq. (1), the cooked break rate was determined for a sample of rice noodles with lengths longer than or equal to 7.5 cm and shorter than 7.5 cm.


(1) $$\mathrm{Cooked}\,\mathrm{broken}\,\mathrm{rate}=\frac{m_{1}}{m_{1}+m_{2}}\times 100\%$$

Where m_1_: after cooking, the weight of semi-dried rice noodles that are shorter than 7.5 cm; m_2_: after cooking, the weight of semi-dried rice noodles that are longer than or equal to 7.5 cm.

#### 2.10.2. Cooking loss rate and rehydration ratio

The method of Cham et al. (23) was modified to assess the cooking loss and rehydration ratio of semi-dried rice noodles. We selected 10 semi-dried rice noodles of about 20 cm in length and weighed m_0_. Rice noodles were put in a beaker, filled with boiling water, boiled for the recommended amount of time (2 min and 30 s), then drained and weighed m_1_. Cooked rice noodles were put on a dry dish and dried at 105°C to a constant weight m_2_, and calculated the cooking loss and rehydration ratio according to the Eqs. (2, 3).


(2) $$\mathrm{Cooking}\,\mathrm{loss}\,\mathrm{rate}=1-\frac{m_{2}}{m_{0}\,\left(1-m\right)}\times 100\%$$

(3) $$\mathrm{Rehydration}\,\mathrm{ratio}=\frac{m_{1}-m_{0}}{m_{0}}\times 100\%$$

Where m: the amount of water in semi-dried rice noodles; m_0_: weight of uncooked semi-dried rice noodles; m_1_: weight of cooked semi-dried rice noodles; m_2_: dry weight of cooked semi-dried rice noodles.

#### 2.10.3. Iodine blue value

With a few modifications from Nagano et al. (24) methods, the turbidity of rice soup with semi-dried rice noodles was measured. We selected 5 semi-dried rice noodles of about 10 cm in length into a beaker, added boiling water, and heated them for 3 min. then rapidly removed the rice noodles, cooled the rice soup, and centrifuged for 5 min at a high-speed refrigerator centrifuge (Centrifugal force of 2,650 *g*). Take 5 mL of supernatant, add 50 mL of distilled water, 1 mL each of 1 mol/L hydrochloric acid, and 1 mL of 0.1 mol/L iodine reagent, and fix the volume. At 620 nm, the absorbance of rice soup was quantified. Add 1 mL of 1 mol/L hydrochloric acid, 1 mL of 0.1 mol/L iodine reagent, and a set volume for the control group.

### 2.11. Textural properties

We examined the texture properties of semi-dried rice noodles samples with a texture analyzer (TA. XT plus C, Stable Micro Systems, London, United Kingdom) following the description given by Li et al. (25) with a few modifications. A total of 15 semi-dried rice noodles were selected, each measuring around 20 cm in length. They were steamed for the optimal steaming time (2 min and 30 s) and then allowed to sit in the test sieve for 5 min. Five semi-dried rice noodles, about 5 cm long, were selected and placed on the texture meter. The settings were model type P/36R, pre-test rate of 2 mm/s, mid-test rate of 1 mm/s, post-test rate of 1 mm/s, a compression ratio of 50%, and compression interval of 3 s.

### 2.12. Color changes

We examined the color changes of semi-dried rice noodles samples with a colorimeter (UltraScan PRO instrument, HunterLab Instruments Inc., USA). According to the method of Wang et al. (26) with minor modifications, we selected 15 semi-dried rice noodles that were about 20 cm long, arranged them neatly, and put them in the colorimeter for photography, mostly referring to L* values and b* values.

### 2.13. Sensory evaluation

The sensory evaluation of semi-dried rice noodles was evaluated according to the method of Aydin et al. (27) with minor modifications. By inviting 10 trained panelists (5 male and 5 female) from the Food Science and Technology Institute were selected for sensory testing. The sensory panelists were trained in the ISO sensory analysis standard (ISO 8586:2012) and pretested using the method described in ISO 11132:2021. A lunch box containing distilled water and 10 semi-dried rice noodles from the sample was filled with the rice noodles after they had been steamed following the optimal steaming time and collected in a beaker. In Supplementary Table 1, the semi-dried rice noodle sensory assessment standard is presented.

### 2.14. X-ray diffraction

An X-ray diffractometer (D8 Advance, Bruker AXS Inc., Karlsruhe, Germany) was used to evaluate the crystallinity of semi-dried rice noodles samples. The diffraction scan region was set at 5° to 50°, the voltage was set at 40 kV, the current was set at 40 mA, and the scan speed was set at 2°/min by the approach given by Tao et al. (15) with minor modifications. Additionally, samples of semi-dried rice noodles were dried at 50°C for 7 h before being pulverized and sieved through sieves with a mesh size of 200.

### 2.15. Moisture distribution

Semi-dried rice noodles samples were subjected to moisture distribution analysis using an NMR analyzer (NMI20, Newmax Analytical Instruments, Suzhou, China), with minor changes to the procedure outlined by Sangpring et al. (28). Before inserting the test tube into the NMR analyzer, we take 10 semi-dried rice noodles, each measuring about 3 cm in length, but then in the test tube to prevent moisture evaporation. The parameters were sampling points TD = 150030, a number of repetitions NS = 32, and TW = 2,000 ms.

### 2.16. Scanning electron microscope (SEM)

The morphological structure of the cross-section of the semi-dried rice noodles samples was examined using a scanning electron microscope (MIRA LMS, Teescan Inc., Brno, Czechia). Semi-dried rice noodles were freeze-dried following the Geng et al. (29) with minor modifications. The samples were then fastened to a sample holder with double-sided carbon tape, coated with gold for 120 s using a sputter coater, and pictures were captured at 3.0 kV accelerating voltage and 1,200× magnification.

### 2.17. Statistical analysis

Using SPSS 20.0, we performed a one-way analysis of variance (ANOVA) (SPSS Inc., Chicago, USA). The measurement for statistical significance was *p* < 0.05. Data were presented using the mean ± standard deviation (*n* = 3). ORIGIN 2018 (Northampton, MA, USA) and GraphPad Prism 8 were used to analyze spectral pictures (GraphPad Software, San Diego, USA).

## 3. Results and discussion

### 3.1. Basic physicochemical composition

Table 1 showed the effect of sodium bicarbonate on the basic physicochemical composition of rice flour. The pH increased with increasing sodium bicarbonate addition, whereas lipid and protein levels decreased. The pH increased from 4.28 to 9.43, while the lipid content and protein content decreased from 0.57 and 7.64 to 0.46 and 5.71%, respectively. The decreased in lipid content was similar to the extraction from soap, and alkali and lipids may interact to generate soap through a process called saponification (7). The decreased in protein content was comparable to the starch extraction that used alkali to remove proteins, and the proteins that were partially eliminated were albumin and globulin, which are soluble in water and alkali solutions, respectively (30). With the addition of sodium bicarbonate from 0 to 0.2%, the amylose content increased, this might be due to the increased removal of protein-bound by starch granules, which increased amylose content (31). With more addition (0.2∼0.5%), the amylose content decreased. This might be because the pH was weakly alkaline, which made the starch and protein partially negatively charged, and the ionic repulsion between negative charges would be broken the hydrogen bond between starch molecules and resulted in the decrease of amylose content (32).

**TABLE 1:** Effect of sodium bicarbonate on the basic physicochemical composition of rice flour.

Addition/%	pH value	Lipid content/%	Protein content/%	Amylose content/%
0	4.28 ± 0.03^h^	0.57 ± 0.01^a^	7.64 ± 0.19^a^	20.04 ± 0.15^e^
0.02	4.53 ± 0.02^g^	0.54 ± 0.01^b^	7.17 ± 0.10^b^	20.43 ± 0.08^d^
0.06	4.78 ± 0.05^f^	0.53 ± 0.01^bc^	7.06 ± 0.05^b^	20.69 ± 0.03^c^
0.1	5.26 ± 0.05^e^	0.52 ± 0.01^c^	6.96 ± 0.04^b^	21.23 ± 0.10^b^
0.2	6.29 ± 0.05^d^	0.51 ± 0.00^d^	6.66 ± 0.20^c^	21.90 ± 0.12^a^
0.3	7.49 ± 0.08^c^	0.50 ± 0.01^d^	6.41 ± 0.02^d^	19.48 ± 0.13^f^
0.4	8.36 ± 0.10^b^	0.47 ± 0.01^e^	6.22 ± 0.13^d^	18.75 ± 0.29^g^
0.5	9.43 ± 0.07^a^	0.46 ± 0.00^f^	5.71 ± 0.20^e^	17.80 ± 0.12^h^

Values are presented as the mean ± SD of three replicate samples. This means in a column that shares the same letters do not significantly differ (*p* < 0.05).

### 3.2. Farinograph properties

Table 2 showed the effect of sodium bicarbonate on the farinograph properties of rice flour. With the addition of sodium bicarbonate was increased, the WA, DDT, and DST of rice flour increased from 55.33%, 0.73 and 0.40 min to 78.33%, 2.17 and 1.30 min, respectively. Consistent with the results of Guo et al. (33), the addition of alkali constantly enhanced dough water absorption. This is due to the fact that aqueous alkali solutions are potent electrolytes and improve the solubility of proteins by exposing their hydrophilic groups, which causes an increase in the water absorption of dough (34). The increase in DDT and DST indicated that the addition of alkali strengthened and increased the stability of the dough, reducing the likelihood that it would break while being mixed (35).

**TABLE 2:** Effect of sodium bicarbonate on the farinograph properties of rice flour.

Addition/%	WA/%	DDT/min	DST/min
0	55.33 ± 0.58^h^	0.73 ± 0.06^f^	0.40 ± 0.00^f^
0.02	59.67 ± 1.15^g^	0.90 ± 0.10^e^	0.43 ± 0.06^ef^
0.06	63.00 ± 1.00^f^	1.13 ± 0.06^d^	0.47 ± 0.06^ef^
0.1	65.00 ± 1.00^e^	1.23 ± 0.06^d^	0.58 ± 0.06^de^
0.2	69.67 ± 1.15^d^	1.53 ± 0.06^c^	0.63 ± 0.06^cd^
0.3	72.67 ± 1.15^c^	1.73 ± 0.12^b^	0.77 ± 0.06^bc^
0.4	75.33 ± 0.58^b^	1.83 ± 0.06^b^	0.83 ± 0.06^b^
0.5	78.33 ± 0.58^a^	2.17 ± 0.15^a^	1.30 ± 0.20^a^

Values are presented as the mean ± SD of three replicate samples. This means in a column that shares the same letters do not significantly differ (*p* < 0.05). WA, dough water absorption; DDT, dough development time; DST, dough stability time.

### 3.3. Pasting properties

Table 3 showed the effect of sodium bicarbonate on the pasting properties of rice flour. Pasting viscosity reflects the degree of swelling of starch granules during pasting (36). With the addition of sodium bicarbonate from 0 to 0.1%, the peak, trough, and final viscosity of semi-dried rice noodles increased; at 0.1 to 0.5%, it decreased. These are due to the breakage of the starch molecular chains brought on by the adsorption of sodium bicarbonate in the amorphous region of the starch granules and the increased swelling of the starch granules (37). The decrease in pasting viscosity was attributable to the further addition of sodium bicarbonate (0.1∼0.5%), since the interaction of sodium ions with hydroxyl groups in starch weakened hydrogen bonds between starch molecules and prevented starch granule expansion (38). A higher peak, trough, and final viscosity contributed to a smooth surface and a good flavor of cooked rice noodles (39).

**TABLE 3:** Effect of sodium bicarbonate on the pasting properties of rice flour.

Addition/%	Peak viscosity/cp	Trough viscosity/cp	Final viscosity/cp	Breakdown/cp	Setback/cp
0	1266.67 ± 38.08^de^	849.33 ± 20.43^bc^	1641.67 ± 60.19^d^	417.33 ± 17.67^b^	792.33 ± 40.05^cd^
0.02	1311.33 ± 12.34^cd^	867.00 ± 13.08^bc^	1703.33 ± 40.61^c^	444.33 ± 5.13^ab^	836.33 ± 27.97^c^
0.06	1372.67 ± 31.90^b^	893.67 ± 17.56^b^	1777.00 ± 5.57^b^	479.00 ± 22.11^a^	883.33 ± 12.01^b^
0.1	1463.00 ± 28.16^a^	975.00 ± 47.84^a^	1908.33 ± 34.79^a^	488.00 ± 19.70^a^	933.33 ± 27.50^a^
0.2	1321.67 ± 33.13^c^	890.33 ± 31.37^b^	1722.33 ± 29.14^bc^	431.33 ± 47.61^b^	832.00 ± 29.31^c^
0.3	1281.33 ± 6.11^cde^	855.67 ± 10.79^bc^	1628.67 ± 4.73^d^	425.67 ± 14.01^b^	773.00 ± 6.08^d^
0.4	1243.67 ± 2.08^e^	825.67 ± 6.43^cd^	1487.33 ± 19.63^e^	418.00 ± 4.58^b^	661.67 ± 24.19^e^
0.5	1165.67 ± 34.43^f^	799.00 ± 11.53^d^	1417.33 ± 34.43^f^	366.67 ± 35.53^c^	618.33 ± 27.10^e^

Values are presented as the mean ± SD of three replicate samples. This means in a column that shares the same letters do not significantly differ (*p* < 0.05).

### 3.4. Thermal properties

Table 4 showed the effect of sodium bicarbonate on the thermal properties of rice flour. The T_0_, T_*p*_, and T_*c*_ increased with the addition of sodium bicarbonate. T_0_, T_*p*_, and T_*c*_ increased from 70.14, 76.70, and 80.95 to 73.70, 78.12, and 83.44, respectively. These were probably caused by the rearrangement of the starch chains following the addition of sodium bicarbonate, which produced a more ordered structure of the starch molecules and helped the thermal stability of crystals, raising the pasting temperature of rice flour (40). Studies showed that the enthalpy of thermal absorption (ΔH) is directly proportional to the degree of starch aging (41). With the addition of sodium bicarbonate from 0 to 0.1%, the ΔH increased; at 0.1 to 0.5 %, it decreased. These were most likely attributable to the fact that sodium bicarbonate disrupted the amorphous region of starch, and reduced the inhibition of straight-chain starch, it would have contributed to the short-term aging of some straight-chain starch in rice flour (42). With more addition (0.1∼0.5%), the starch’s hydroxyl groups interacted with sodium ions to decrease water mobility in the starch-water system and inhibited the aging of starch (43).

**TABLE 4:** Effect of sodium bicarbonate on the thermal properties of rice flour.

Addition/%	T_0_/°C	T_*p*_/°C	T_*c*_/°C	Δ H/J⋅g^–1^
0	70.14 ± 0.09^g^	76.70 ± 0.00^g^	80.95 ± 0.05^h^	2.69 ± 0.13^f^
0.02	71.23 ± 0.16^f^	76.95 ± 0.00^f^	81.40 ± 0.05^g^	3.18 ± 0.00^e^
0.06	72.28 ± 0.05^e^	77.10 ± 0.12^ef^	81.83 ± 0.00^f^	3.91 ± 0.02^b^
0.1	72.68 ± 0.09^d^	77.16 ± 0.03^e^	82.10 ± 0.00^e^	4.44 ± 0.07^a^
0.2	72.95 ± 0.00^c^	77.37 ± 0.03^d^	82.58 ± 0.05^d^	3.99 ± 0.02^b^
0.3	73.22 ± 0.00^b^	77.53 ± 0.05^c^	82.82 ± 0.09^c^	3.78 ± 0.04^c^
0.4	73.34 ± 0.05^b^	77.76 ± 0.15^b^	83.10 ± 0.00^b^	3.67 ± 0.03^d^
0.5	73.70 ± 0.10^a^	78.12 ± 0.20^a^	83.44 ± 0.12^a^	3.09 ± 0.02^e^

Values are presented as the mean ± SD of three replicate samples. This means in a column that shares the same letters do not significantly differ (*p* < 0.05). T*_o_*, onset temperatures of thermal; T*_p_*, peak temperatures of thermal; T*_c_*, conclusion temperatures of thermal; ΔH, enthalpy changes of thermal.

### 3.5. Rheological properties

#### 3.5.1. Static rheological

**FIGURE 1:**
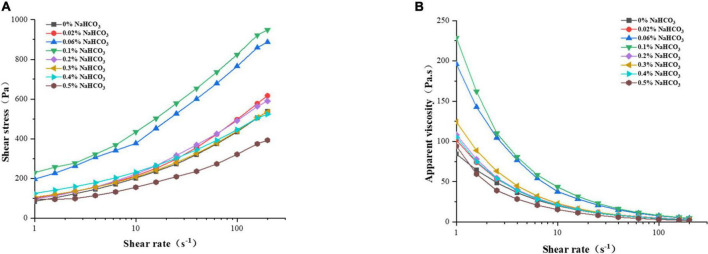
Effect of sodium bicarbonate on shear stress **(A)** and apparent viscosity **(B)** of rice flour.

The effect of sodium bicarbonate on the static rheological properties of rice flour was shown in Figure 1. As shown in Figure 1A, as the addition of sodium bicarbonate was from 0 to 0.1%, the shear stress of rice flour increased; and it decreased at 0.1 to 0.5 %. Chen reported that the higher shear stress of rice flour indicates the higher gel strength and more stable gel structure of rice noodles (44). The maximum shear stress of rice flour at 0.1% addition of sodium bicarbonate indicated the maximum gel strength and the most stable gel structure of semi-dried rice noodles. As shown in Figure 1B, the apparent viscosity of rice flour gradually decreased with the increased shear rate, which has the characteristic of shear thinning and typical of non-Newtonian fluids (45).

#### 3.5.2. Dynamic rheological

**FIGURE 2:**
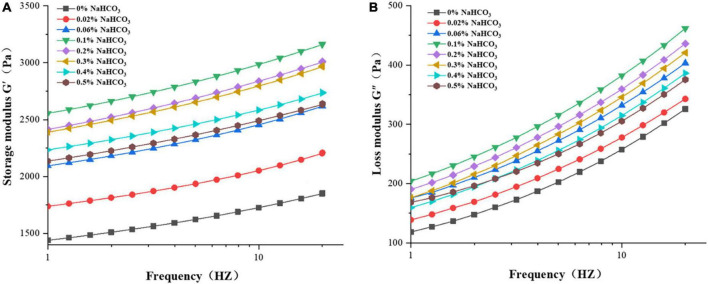
Effect of sodium bicarbonate on storage modulus **(A)** and loss modulus **(B)** of rice flour.

The effects of sodium bicarbonate on the dynamic rheological properties of rice flour were shown in Figure 2. The storage modulus (G′) and loss modulus (G″) of rice flour increased with the increased frequency, and G′ was higher than G″ it demonstrated that more elastic characteristics were exhibited of rice flour. As the addition of sodium bicarbonate was from 0 to 0.1%, the G′and G″ increased; at 0.1 to 0.5%, it decreased. According to Meerts et al. (46), the rheological properties of dough were significantly impacted by the interaction between starch and molecules. With the addition of a moderate amount of sodium bicarbonate (0∼0.1%) might increase the interaction between sodium ions and starch molecules, and that would contribute to an increase in G′ and G″. This result was in agreement with Beck et al. (47). With more addition (0.1∼0.5%),with increased addition, excessive sodium ions affected on gel structure of rice flour and resulted in decreased G′ and G″ (48).

### 3.6. Cooking properties

Table 5 showed the effect of sodium bicarbonate on the cooking properties of semi-dried rice noodles. Cooking properties were influenced by cooked broken rate, cooking loss, and rehydration. The turbidity of the cooked rice soup was represented in the iodine blue value. Cooking loss, the ratio of rehydration, and the value of iodine blue all rose with the addition of more sodium bicarbonate, whereas cooked broken rate initially decreased and subsequently increased. The cooked broken rate of semi-dried rice noodles decreased to the lowest rate of 1.02% with the addition of sodium bicarbonate at a rate of 0.1%. These are most likely attributed by sodium bicarbonate causing hydrogen bonds between starch molecules to break, which made the gel structure of semi-dried rice noodles more stable and decreased the cooked broken rate. As the amount added increased (0.1∼0.5%), the interaction of sodium ions with hydroxyl groups in starch reduced the interaction of water with starch, and made the gel structure of semi-dried rice noodles looser, and caused increased cooked broken rate (40, 43). Cooking loss and iodine blue value increased with the addition of sodium bicarbonate from 8.18% and 0.281 to 13.51% and 0.532, respectively. This could be explained by sodium bicarbonate’s disruption of the amorphous region of starch molecules, which promoted starch dissolution and led to some straight-chain starch inside the starch dissolving out and dissolving in rice soup when semi-dried rice noodles were steamed (23). In addition to the fact that the addition of sodium bicarbonate increased the rehydration ratio of semi-dried rice noodles, it is likely that this phenomenon was caused by the breaking of hydrogen bonds in the amorphous region, which led to slow and irreversible water absorption (42).

**TABLE 5:** Effect of sodium bicarbonate on the cooking properties of semi-dried rice noodles.

Addition/%	Cooked broken rate/%	Cooking loss rate/%	Rehydration ratio/%	Iodine blue value
0	2.56 ± 0.28^e^	8.18 ± 0.44^g^	69.14 ± 1.79^h^	0.281 ± 0.003^g^
0.02	1.86 ± 0.41^f^	8.79 ± 0.03^f^	72.06 ± 0.93^g^	0.285 ± 0.002^g^
0.06	1.21 ± 0.14^g^	9.11 ± 0.10^ef^	74.55 ± 0.54^f^	0.290 ± 0.003^f^
0.1	1.02 ± 0.12^g^	9.31 ± 0.04^e^	76.07 ± 0.24^e^	0.296 ± 0.002^e^
0.2	3.76 ± 0.26^d^	10.19 ± 0.16^d^	78.20 ± 0.16^d^	0.316 ± 0.003^d^
0.3	5.38 ± 0.18^c^	10.64 ± 0.21^c^	80.75 ± 0.22^c^	0.477 ± 0.001^c^
0.4	6.59 ± 0.04^b^	12.23 ± 0.11^b^	84.43 ± 1.13^b^	0.487 ± 0.005^b^
0.5	8.47 ± 0.44^a^	13.51 ± 0.30^a^	88.70 ± 0.35^a^	0.532 ± 0.004^a^

Values are presented as the mean ± SD of three replicate samples. This means in a column that shares the same letters do not significantly differ (*p* < 0.05).

### 3.7. Textural properties

Table 6 showed the effect of sodium bicarbonate on the textural properties of semi-dried rice noodles. Hardness is a vital parameter for cooking properties and chewiness affects consumer preferences (49). With the addition of sodium bicarbonate from 0 to 0.1%, the hardness and chewiness of semi-dried rice noodles increased; at 0.1 to 0.5%, it decreased. According to Tang et al. (50), the aging of starch was positively connected with the hardness of rice noodles. These could be attributed to sodium bicarbonate disrupting the amorphous region of starch, and the reduction of the amorphous region accelerated the short-term aging of some straight-chained starches, increased the orderly structure, and recrystallization formed by starch molecules, and increased the hardness of semi-dried rice noodles (42). With more addition (0.1∼0.5%), the interaction of too many sodium ions with the hydroxyl groups in starch decreased the interaction between water and starch, prevented the aging of starch, and disorganized the microcrystalline structure formed during the recrystallization of starch, resulting in a decrease in the hardness of semi-dried rice noodles (43). According to studies, the chewiness and hardness of rice noodles have a significant impact on their taste, with moderate hardness and high chewiness producing the tastiest rice noodles (51).

**TABLE 6:** Effect of sodium bicarbonate on the textural properties of semi-dried rice noodles.

Addition/%	Hardness/g	Adhesiveness/g⋅s	Resilence/%	Cohesion	Springiness/%	Chewiness/g
0	3140.56 ± 85.26^bc^	−32.90 ± 6.64^b^	46.86 ± 2.94^ab^	0.70 ± 0.04^ab^	87.91 ± 1.80^ab^	1932.89 ± 142.86^a^
0.02	3166.16 ± 121.63^b^	−19.57 ± 9.34^a^	49.31 ± 1.45^a^	0.74 ± 0.02^a^	86.40 ± 1.62^b^	1957.67 ± 44.04^a^
0.06	3258.59 ± 158.38^b^	−24.53 ± 7.02^ab^	45.39 ± 2.31^b^	0.72 ± 0.01^a^	86.54 ± 3.16^b^	1972.68 ± 63.42^a^
0.1	3470.86 ± 128.15^a^	−27.49 ± 7.09^ab^	44.44 ± 1.59^bc^	0.67 ± 0.02^bc^	86.80 ± 1.47^b^	2010.31 ± 38.60^a^
0.2	2958.31 ± 104.32^cd^	−16.48 ± 7.32^a^	41.71 ± 1.07^c^	0.64 ± 0.02^c^	86.48 ± 2.66^b^	1861.72 ± 67.19^a^
0.3	2777.18 ± 106.16^de^	−17.05 ± 3.77^a^	43.65 ± 0.79^bc^	0.73 ± 0.00^a^	91.68 ± 0.70^a^	1683.17 ± 134.48^b^
0.4	2594.64 ± 38.33^e^	−15.66 ± 2.25^a^	44.49 ± 1.66^bc^	0.74 ± 0.03^a^	87.52 ± 3.23^b^	1639.24 ± 92.04^b^
0.5	2361.53 ± 109.23^f^	−26.78 ± 4.04^ab^	37.73 ± 2.27^d^	0.64 ± 0.03^c^	85.78 ± 1.94^b^	1288.97 ± 48.67^c^

Values are presented as the mean ± SD of three replicate samples. This means in a column that shares the same letters do not significantly differ (*p* < 0.05).

### 3.8. Color changes

Table 7 showed the effect of sodium bicarbonate on the color of semi-dried rice noodles. The L* values decreased from 81.71 to 72.64 and b* values increased from 8.10 to 12.85 as the amount of sodium bicarbonate added increased. This demonstrated that the semi-dried rice noodles had a yellow color when made with the addition of sodium bicarbonate and that the color of the semi-dried rice noodles grew with the addition of sodium bicarbonate. This modification might be explained by the fact that the raw materials’ flavonoid compounds are released with the increasing addition of sodium bicarbonate, giving semi-dried rice noodles their yellow appearance (52).

**TABLE 7:** Effect of sodium bicarbonate on the color changes of semi-dried rice noodles.

Addition/%	Appearance	L*	a*	b*
0	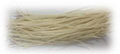	81.71 ± 0.67^a^	0.07 ± 0.02^e^	8.10 ± 0.51^f^
0.02	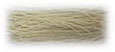	80.13 ± 0.47^b^	0.10 ± 0.01^d^	8.89 ± 0.16^e^
0.06	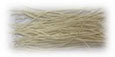	78.19 ± 0.16^c^	0.12 ± 0.01^d^	9.88 ± 0.72^d^
0.1	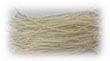	76.71 ± 0.21^d^	0.16 ± 0.03^c^	11.55 ± 0.13^c^
0.2	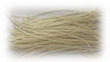	76.35 ± 0.73^d^	0.22 ± 0.00^b^	11.99 ± 0.12^bc^
0.3	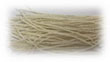	75.96 ± 0.51^de^	0.23 ± 0.01^ab^	12.23 ± 0.19^b^
0.4	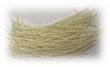	75.39 ± 0.12^e^	0.23 ± 0.00^ab^	12.59 ± 0.14^ab^
0.5	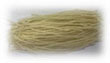	72.64 ± 0.37^f^	0.25 ± 0.01^a^	12.85 ± 0.11^a^

Values are presented as the mean ± SD of three replicate samples. This means in a column that shares the same letters do not significantly differ (*p* < 0.05).

### 3.9. Sensory evaluation

**FIGURE 3:**
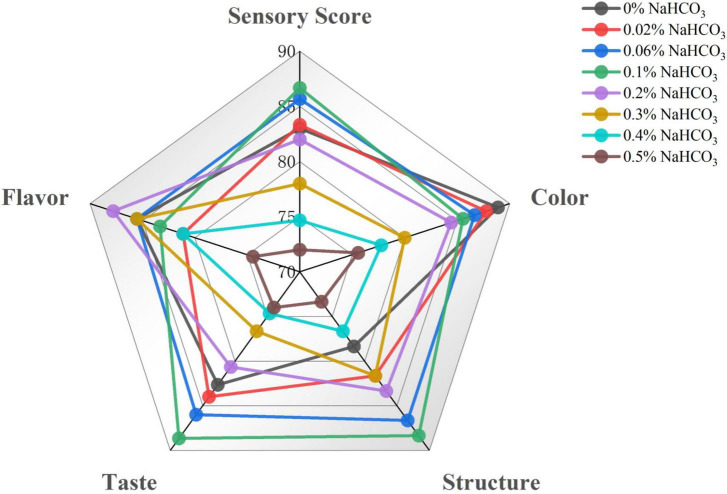
Effect of sodium bicarbonate on the sensory evaluation of semi-dried rice noodles.

The effect of sodium bicarbonate on the sensory evaluation of semi-dried rice noodles was shown in Figure 3. The color of the semi-dried rice noodles made with the addition of sodium bicarbonate was slightly less pleasing, indicating that the addition of sodium bicarbonate produced semi-dried rice noodles with a yellowish tint. The overall sensory evaluation score was higher for semi-dried rice noodles as they displayed superior structural integrity and flavor when sodium bicarbonate was added in the proper proportion. Semi-dried rice noodles had the highest total sensory evaluation score of 86.67 in addition to the 0.1% sodium bicarbonate. This outcome could be explained by the important roles that rice noodle structure and flavor played.

### 3.10. Crystallinity

**FIGURE 4:**
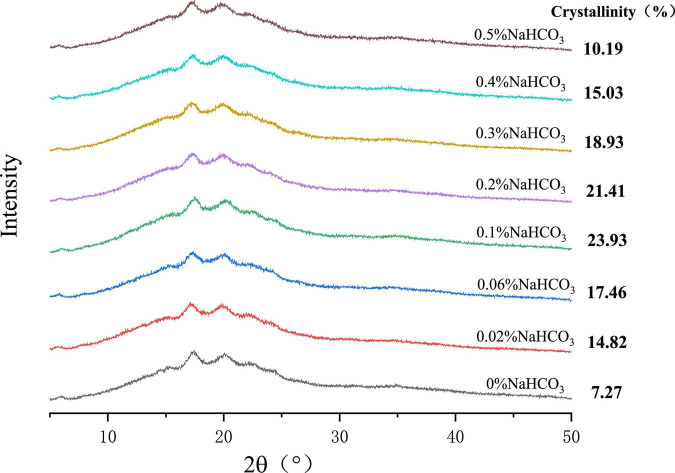
Effect of sodium bicarbonate on the crystallinity of semi-dried rice noodles.

The effect of sodium bicarbonate on the crystallinity of semi-dried rice noodles was shown in Figure 4. All of the semi-dried rice noodle samples had typical B-type crystals with typical diffraction peak sites at around 5.6°, 17°, 20°, and 22°. Since these peak locations corresponded to the normal peaks of starch aging, it was clear that the aging of the semi-dried rice noodle samples exhibited aging of starch. The outcomes demonstrated that the addition of sodium bicarbonate would not change the starch’s crystal structure, which was consistent with Wang and Tao’s (15, 53) report that the alkali treatment did not affect starch’s crystal structure. With the addition of sodium bicarbonate from 0 to 0.1%, the crystallinity increased; at 0.1 to 0.5%, it decreased. These might be related to the sodium bicarbonate-induced breakdown of the amorphous sections of the starch molecules and the orderly stacking of double helices of starch chains to generate more ordered structures and increase crystallinity (54). The decreased crystallinity was due to the further addition of sodium bicarbonate (0.1∼0.5%), the interaction of sodium ions with hydroxyl groups in starch prevented the formation of hydrogen bonds between them, which resulted in the disordering of starch microcrystals and the reduction or unraveling of the tightness in the double helix (28). According to studies, starch-based foods’ crystallinity is a key measure of their quality, and it is directly correlated with the products’ hardness, and moisture distribution (55).

### 3.11. Moisture distribution

**FIGURE 5:**
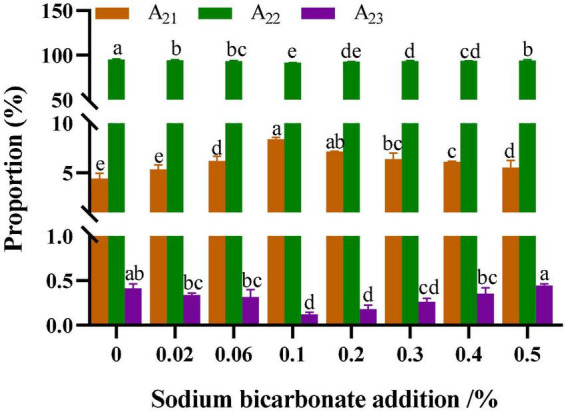
Effect of sodium bicarbonate on the moisture distribution of semi-dried rice noodles. Means of the same parameter with different superscript letters indicate significant difference (*p* < 0.05).

The effect of sodium bicarbonate on the moisture distribution of semi-dried rice noodles was shown in Figure 5. As shown in Figure 4, the bound water, weakly bound water, and free water contents in semi-dried rice noodles were A_21_, A_22_, and A_23_, respectively. With the addition of sodium bicarbonate from 0 to 0.1%, A_21_ increased, A_22_ and A_23_ decreased in semi-dried rice noodles; at 0.1 to 0.5%, A_21_ decreased, and A_22_ and A_23_ increased. These could be attributed to sodium bicarbonate disrupting the amorphous region of starch, which facilitates some straight-chain starch recrystallizing with water molecules and promoted the interaction between starch chains and water molecules, resulting in an increase in the amount of bound water in the semi-dried rice noodles (42). With more being added (0.1∼0.5%), the sodium ion concentration increased. The interaction of sodium ions with the hydroxyl groups in starch inhibited the recrystallization of starch and water molecules, decreased the quantity of water diffusing from the amorphous to the crystalline area, and increased the mobility of water in semi-dried rice noodles (43). Singh said that higher amounts of bound water meant less water that wasn’t bonded firmly and had a higher water-holding capacity (56).

### 3.12. Morphological characteristics

**FIGURE 6:**
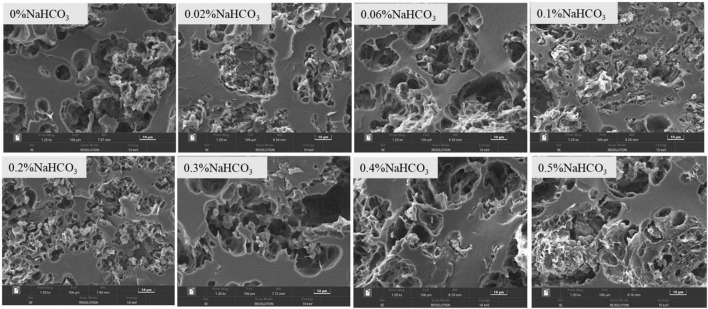
Effect of sodium bicarbonate on the microscopic morphology of semi-dried rice noodles.

The effect of sodium bicarbonate on the microscopic morphological structure of semi-dried rice noodles was shown in Figure 6. With the addition of sodium bicarbonate from 0 to 0.1%, the starch-protein combination increased, starch granules were embedded in the protein network structure, and starch and protein were formed into an orderly and stable network structure. With the further addition of sodium bicarbonate (0∼0.5%), there were fewer agglomerated structures, the starch-protein network structure was distorted, and starch granules could not be properly embedded in the protein network structure. This showed that lower sodium bicarbonate concentrations caused greater interaction between the starch and protein in semi-dried rice noodles and totally contained the starch granules within the protein network structure. However, excessive sodium bicarbonate would make the starch-protein network structure lose and discontinuous, and result in the separation of starch granules from the network structure.

### 3.13. Principal component analysis (PCA)

**FIGURE 7:**
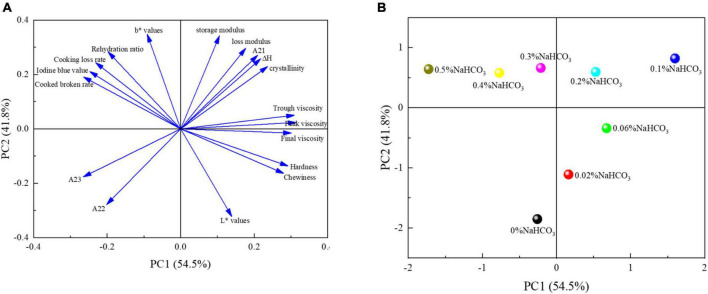
Principal component analysis of semi-dried rice noodles with different sodium bicarbonate additions. Loading plot of the variables of samples **(A)**. Score plot of the different samples **(B)**.

The principal component analysis of semi-dried rice noodles with different sodium bicarbonate additions was shown in Figure 7. As shown in Figure 7A, the loading plots clearly demonstrated that PC1 and PC2 contributed 96.3% of the variance, with respective variance contributions of 41.8 and 54.5%. Therefore, the PC1-PC2 plane could represent the response variables’ primary contribution. With various amounts of sodium bicarbonate, PCA score plots showed that the quality of semi-dried rice noodles dramatically altered. As shown in Figure 7B, the addition of 0.1% sodium bicarbonate produced semi-dried rice noodles with the best quality and overall scores. In light of this, it may be said that adding a small amount of sodium bicarbonate could enhance the chewiness, flavor, and edible quality of semi-dried rice noodles.

## 4. Conclusion

The addition of sodium bicarbonate had a significant effect on the physicochemical properties of rice flour and the quality characteristics of semi-dried rice noodles. With the increase of sodium bicarbonate addition, the pH value was increased, and lipid and protein content were decreased in rice flour. Meanwhile, Thermal and farinograph properties showed that the pasting temperature, dough water absorption, dough development time, and dough stability time of rice flour increased with the addition of sodium bicarbonate. Pasting and rheological properties of rice flour revealed that a modest amount of sodium bicarbonate could be increased the pasting viscosity, storage modulus (G’), and loss modulus (G″), and that the G′ were more than G″ for all samples, it demonstrated that rice flour showed more elastic characteristics. Additionally, with the addition of sodium bicarbonate from 0 to 0.1%, the cooked broken rate decreased, and the hardness and chewiness of semi-dried rice noodles increased. With the addition of a small amount of sodium bicarbonate, X-ray diffraction showed that it could be increased the crystallinity of semi-dried rice noodles, which increased from 7.27 to 23.93%, it demonstrated that the addition of sodium bicarbonate could increase the short-term aging of starch. Low-field nuclear magnetic resonance showed that A_21_ increased, A_22_ and A_23_ decreased in semi-dried rice noodles, A_21_ increased from 4.44 to 8.39%, A_22_ and A_23_ decreased from 95.16 and 0.41 to 91.48 and 0.12%, respectively. Scanning electron microscope showed that it could increase the starch-protein interaction and result in the formation of an ordered and stable network structure of starch-protein. Finally, the principal component analysis showed that the chewiness, texture and eating quality of semi-dried rice noodles were the best with the addition of sodium bicarbonate at 0.1%. Although this study elucidated the significant effects of sodium bicarbonate on the physicochemical properties of fermented rice flour and the quality characteristics of fermented semi-dried rice noodles, the variation of acidic taste in fermented semi-dried rice noodles was not specifically analyzed by instrumentation. Further studies are needed to accurately analyze the dynamics of acidic taste and flavor in fermented semi-dried rice noodles made with different sodium bicarbonate additions using electronic nose or gas chromatography-mass spectrometry (GC-MS).

## Data availability statement

The original contributions presented in this study are included in this article/Supplementary material, further inquiries can be directed to the corresponding author.

## Author contributions

LL and WX devised and planned the experiments. YD, WX, and YC carried out the experiments. WX, YC, and SX examined the data. WX and YD wrote the manuscript. All authors have been reviewed and approved the final draft.
