# Supplementary material for: Effect of sodium bicarbonate on the physicochemical properties of fermented rice flour and quality characteristics of fermented semi-dried rice noodles
Source: Front Nutr. 2023 Feb 16;10:1100422. doi: 10.3389/fnut.2023.1100422 (PMC9978011; doi:10.3389/fnut.2023.1100422)
Supplement: Supplementary file 1 [file Table_1.DOCX]

Table S1 Sensory evaluation table of semi-dried rice noodles

| Sensory indicators | Item rating | Score |
| --- | --- | --- |
| Color  (20 points) | Distinctive beige color, good transparency | 16~20 |
|  | Normal color, good transparency | 11~15 |
|  | Yellowish color, poor transparency | 0~10 |
| Structure  (20 points) | Smooth surface, no broken strips | 16~20 |
|  | Smooth surface, less broken strips | 11~15 |
|  | Surface unsmooth, more broken strips | 0~10 |
| Flavor  (20 points) | Fermented rice with strong aroma and without odor | 16~20 |
|  | Fermented rice fragrance, slight odor | 11~15 |
|  | No fermented rice aroma, serious odor | 0~10 |
| Taste  (40 points) | Soft and smooth, no sticky teeth, no raw material | 31~40 |
|  | Softer and smoother, with sticky teeth or raw material | 21~30 |
|  | Poor taste, with sticky teeth or raw material | 0~20 |
